# Attention Xception U‐Net (AXUNet): An explainable combination of CNN and self‐attention for reliable glioma segmentation in MRI

**DOI:** 10.1002/acm2.70902

**Published:** 2026-11-13

**Authors:** Farzan Moodi, Fereshteh Khodadadi Shoushtari, Gelareh Valizadeh, Dornaz Mazinani, Hanieh Mobarak Salari, Sedigheh Sina, Hamidreza Saligheh Rad

**Affiliations:** ^1^ Quantitative MR Imaging and Spectroscopy Group (QMISG) Tehran University of Medical Sciences Tehran Iran; ^2^ Department of Radiology, Shariati Hospital Tehran University of Medical Sciences Tehran Iran; ^3^ Nuclear Engineering Department Shiraz University Shiraz Iran; ^4^ Radiation Research Center Shiraz University Shiraz Iran; ^5^ Department of Medical Physics and Biomedical Engineering Tehran University of Medical Sciences Tehran Iran

**Keywords:** brain MRI, BraTS, deep learning, explainable AI, glioma segmentation, self‐attention, UNet

## Abstract

**Background:**

Gliomas are the most common primary malignant brain tumors and exhibit highly heterogeneous morphology and infiltrative growth patterns. Accurate delineation of tumor subregions in magnetic resonance imaging (MRI) is therefore critical for diagnosis, treatment planning, and longitudinal monitoring, yet manual segmentation remains time‐consuming and subject to inter‐observer variability.

**Purpose:**

Accurate glioma segmentation in magnetic resonance imaging (MRI) is essential for diagnosis, treatment planning, and reproducible tumor delineation. However, for clinical adoption, automated segmentation systems must provide not only high accuracy but also robustness and interpretability. This study aimed to develop an explainable and reliable deep learning framework for glioma segmentation and to evaluate its performance on both internal and external datasets.

**Method:**

We used the BraTS 2021 training dataset, which contains 1251 multi‐institutional pre‐operative glioma MRI cases. After comparing multiple sequence combinations, T1ce, T2, and Fluid Attenuated Inversion Recovery (FLAIR) were selected as the final three‐channel input. Preprocessing included tumor‐oriented slice selection, cropping, Min‐Max normalization, and augmentation. We developed AXUNet, a UNet‐based architecture that integrates memory‐efficient Xception blocks with a self‐attention module combining kernel‐based pixel attention and dot‐product channel attention. Internal ablation analysis and Gradient‐weighted Class Activation Mapping (Grad‐CAM)‐based visualization were also performed.

**Results:**

On the BraTS 2021 test partition, AXUNet achieved the highest mean Dice score (93.73), outperforming UNet (93.26), Inception‐UNet (90.88), Attention Gate UNet (AG‐UNet) (90.38), Attention ResUNet (AResUNet) (92.80), and Xception‐UNet (93.24). Region‐wise Dice scores were 92.59 for Whole Tumor (WT), 86.81 for Tumor Core (TC), and 84.89 for Enhancing tumor (ET). Although AResUNet achieved a slightly higher ET score (84.92), AXUNet demonstrated the strongest performance in mean Dice, WT, and TC. To further assess generalizability, AXUNet was independently evaluated on 219 previously unseen BraTS 2023 cases through the Synapse platform, achieving Dice scores of 86.29% for WT, 78.53% for TC, and 69.00% for ET.

**Conclusion:**

AXUNet demonstrates that combining Xception‐based encoding with self‐attention can improve glioma segmentation while also supporting explainability and external robustness, positioning it as a promising framework for MRI‐based glioma segmentation. Its clinical relevance, and the practical value of its interpretability outputs, remain to be established through further, dedicated validation beyond Dice‐based benchmarking.

## 1 INTRODUCTION

Gliomas are the most common group of primary malignant brain tumors, divided into low‐grade gliomas (LGG) consisting of grades 1 and 2, and high‐grade gliomas (HGG) consisting of grades 3 and 4.^1^ High‐grade tumors have a poor prognosis, with patients often surviving less than a year and a half.^2^ Various factors influence patient survival, one of which is tumor boundary, as it determines the area for radiation treatment and surgical resection. Therefore, accurate delineation of glioma boundaries is essential for effective clinical decision‐making and precision medicine.

Traditionally, tumor segmentation has been performed manually by clinicians. However, the results can vary depending on the clinician's expertise, leading to inconsistent outcomes. Moreover, this manual process can be time‐consuming, especially for magnetic resonance imaging (MRI) scans with numerous image slices. These inconsistencies hinder reproducibility and objectivity in treatment planning. Automated segmentation using artificial intelligence (AI) serves as an efficient decision‐support tool by translating expert annotation knowledge into algorithmic decision‐making.^3^, ^4^ Nonetheless, in clinical practice, acceptance of automatic methods depends not only on accuracy but also on transparency and explainability of the AI model to ensure trust.^5^

Initially, machine learning algorithms comprising supervised or unsupervised algorithms were employed for tumor segmentation. However, these methods had their own challenges. Supervised methods required manual feature extraction, while unsupervised methods, such as clustering or seeding techniques, struggled with determining the initial centroid or seed location, presenting significant hurdles for segmentation.^6^ The introduction of the UNet^7^ model revolutionized the field of segmentation, and the Brain Tumor Segmentation Challenge (BraTS)^8^ further drew attention to glioma segmentation.

As deep learning achieved remarkable accuracy, interpretability and ethical accountability have emerged as critical priorities.^9^ Modern clinical AI must not only segment tumors correctly but also justify the reasoning behind each voxel‐level decision. Black‐box CNN models, while effective, lack transparent mechanisms for validation against human judgment.^3^ This gap—between accuracy and explainability—has motivated the development of Explainable Neural Architectures (XNA) that integrate visualization‐based interpretability (e.g., Gradient‐weighted Class Activation Mapping (Grad‐CAM)) and ethical evaluation criteria such as fairness, robustness, and reproducibility.^5^, ^10^

In this study, building upon previous research, we propose the Attention Xception UNet (AXUNet) model that combines the power of UNet architecture with memory‐efficient Xception blocks, and an attention module called self‐attention that gives rise to the state‐of‐the‐art (SOTA) natural language processing models such as OpenAI Chat GPT and Google Gemini.^11^ Taking inspiration from the multi‐attention network (MANet) model^12^ which has shown successful application in Remote Sensing Images, we endeavored to design, implement, and develop our proposed model. These self‐attention modules provide pixel‐wise and channel‐wise adaptability, enabling AXUNet to highlight tumor‐relevant areas while suppressing background features in a clinically meaningful way. From the perspective of ethical AI design, this selective attention supports transparency and fairness by reducing bias toward dominant intensity regions and improving spatial consistency.

Furthermore, we aimed to align AXUNet's development with the principles of Trustworthy AI in Healthcare, as defined by frameworks such as FUTURE‐AI and Healthcare AI Trustworthiness Index (HAITI).^5^, ^13^ Accordingly, we conducted a rigorous internal ablation study on BraTS 2021 Adult Glioma training set to refine module placement and validate performance stability, followed by evaluation on the hidden BraTS 2023 validation cohort on 219 cases in BraTS 2023 Adult Glioma validation set through the Synapse platform. This independent evaluation provides additional evidence of robustness, reliability, and generalizability across previously unseen clinical data, which are essential pillars of ethical AI deployment.^14^

Prior work on segmentation‐network design and attention mechanisms provides important context for these architectural choices. The UNet model pioneered deep learning‐based medical image segmentation and featured a symmetric encoder–decoder architecture.^7^ Its skip connections alleviated information loss but could not fully preserve fine spatial details across multiple down‐sampling layers. Successive convolutions often caused vanishing gradients, impeding the training of deeper models. To address this, ResNet introduced residual blocks, allowing stable gradient propagation and improved convergence.^15^ UNet variants subsequently incorporated these blocks to enhance learning dynamics and feature reuse.

Nevertheless, standard UNet architectures pass all encoder features to the decoder indiscriminately, including irrelevant signals. This unfiltered information transfer may overwhelm the decoder—an issue known as information bombardment. To overcome this, attention mechanisms were introduced,^16^ enabling networks to selectively focus on diagnostically significant regions, such as tumor areas in our case. The concept aligns with explainable AI principles, as it improves both localization performance and interpretability for clinicians by explicitly highlighting model attention.

Attention modules can be classified based on four features^17^:

- The Softness of Attention which is defined as soft (deterministic)/hard (stochastic) or global/local attention.
- Forms of Input Feature categorized as either item‐wise or location‐wise, where the former comprises single items like words and the latter comprises of inputs that are not discrete like images.
- Input Representations which can be categorized as distinctive, self (ours), co‐attention, hierarchical based on the number of inputs.
- Output Representations which can be categorized as single‐output, multi‐head, and multi‐dimensional based on the output of one attention map, multiple attention maps for multiple inputs or multiple attention maps for one input, respectively.

For medical segmentation, attention mechanisms have been strategically inserted at various points in the UNet architecture, each with interpretability trade‐offs. Maji et al.^18^ applied attention gates between skip connections and decoder blocks to guide information filtering. Jia et al.^19^ applied coordinate attention averaging along axial, sagittal, and coronal planes within the decoder, implicitly improving focus consistency. Zhou et al.^20^ proposed attention‐aware multi‐modal fusion using multi‐sized kernels to enhance both spatial and channel awareness. Cao et al.^21^ integrated contextual multiscale CBAM modules with residual UNet to jointly model spatial and channel correlations.

While recent models have improved segmentation accuracy, clinical translation still requires explainability and trust. AXUNet addresses this by integrating self‐attention into a CNN backbone and enabling Grad‐CAM–based visualization of feature relevance, thereby supporting a better balance between performance and interpretability.

## 2 METHOD

### 2.1 Dataset

In this study, we utilized the BraTS 2021 dataset,^22^ a multi‐institutional and multi‐vendor collection of pre‐operative glioma MRI scans. The dataset includes four structural MRI sequences for each subject: T1‐weighted (T1), contrast‐enhanced T1‐weighted (T1ce), T2‐weighted (T2), and Fluid Attenuated Inversion Recovery (FLAIR). A total of 1251 low‐grade (LGG) and high‐grade glioma (HGG) cases were included.

All images were previously co‐registered to a common anatomical template and resampled to 1 mm^3^ isotropic resolution, with spatial dimensions of 155 × 240 × 240 voxels per case. Ground‐truth annotations were initially generated using automated segmentation algorithms and subsequently refined by expert neuroradiologists.

Tumor subregions were defined according to the BraTS protocol as:

- Peritumoral edema (PE),
- Necrotic and non‐enhancing tumor core (NCR/ECT),
- Enhancing tumor (ET).

From these labels, composite regions were derived for evaluation:

- Tumor Core (TC = NCR/ECT + ET),
- Whole Tumor (WT = TC + PE),

while ET was preserved as an independent region.

### 2.2 Preprocessing and data augmentation

The preprocessing workflow is illustrated in Figure 1, including both image preparation (Figure 1(a)) and mask preparation (Figure 1(b)).

**FIGURE 1:**
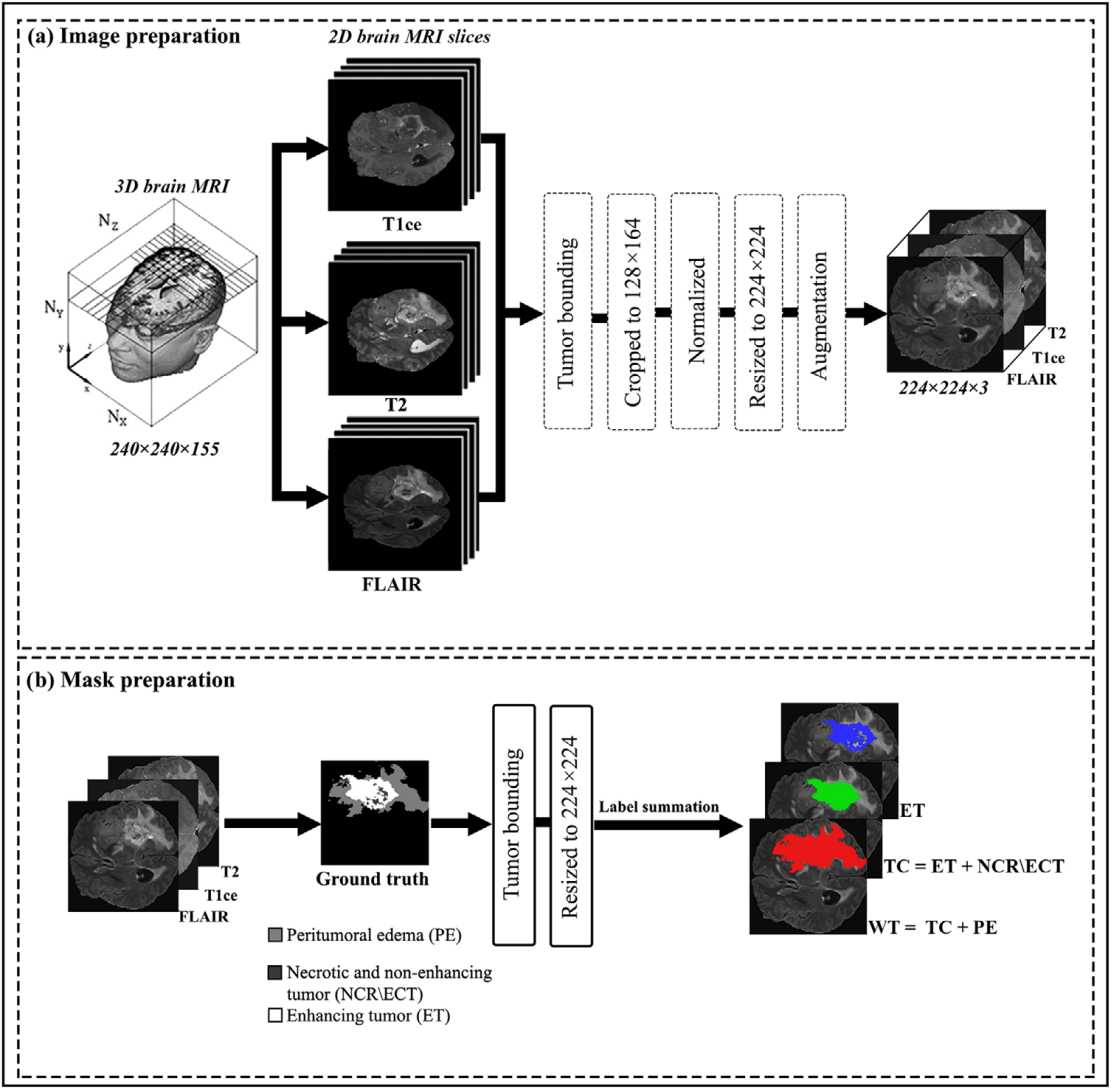
Preprocessing workflow: (a) Image preparation and (b) Mask preparation.

#### 2.2.1 Sequence selection and data split

Multiple combinations of MRI sequences were explored. The combination of T1ce, T2, and FLAIR yielded the best validation performance and was selected for final training. These sequences were concatenated to form three‐channel input tensors.

The dataset was randomly partitioned at the subject level into:

- 80% training,
- 10% validation,
- 10% testing.

#### 2.2.2 Tumor‐oriented slice selection and spatial cropping

To reduce background dominance, only slices containing at least 0.7% tumor pixels were retained. This threshold was adapted from a previous study,^23^ in which an initial threshold of 0.5% was fine‐tuned to 0.7% using a subset of the data, yielding better performance.

As shown in Figure 1(a), non‐informative black regions were removed by cropping images to brain boundaries along height and width axes, resulting in images of size 128 × 164 pixels.

Mask cropping was performed identically to ensure spatial alignment (Figure 1(b)).

This tumor‐aware filtering strategy reduces bias introduced by large homogeneous background regions and improves feature learning efficiency.

#### 2.2.3 Intensity normalization

Due to inter‐institutional variability in MRI acquisition parameters, Min–Max normalization was applied independently to each image channel:

(1) $$I_{norm}=\frac{I-I_{min}}{I_{max}-I_{min}}\;$$

where I denotes original pixel intensity, and $I_{min}$, $I_{max}$ represent the minimum and maximum intensity values within each channel.

As defined in Equation (1), this normalization scales intensities to [0,1], improving numerical stability and cross‐case consistency.

#### 2.2.4 Mask processing

Segmentation masks were processed according to BraTS definitions:

- TC = NCR/ ECT + ET
- WT = TC + PE
- ET unchanged

Tumor bounding was consistently applied to masks to preserve alignment with cropped images (Figure 1(b)).

#### 2.2.5 Data augmentation

To improve generalization, on‐the‐fly augmentation was performed using Albumentations.^24^

Before augmentation, images and masks were resized to 224 × 224 to match the input configuration of the proposed architecture (Figure 2(a)).

**FIGURE 2:**
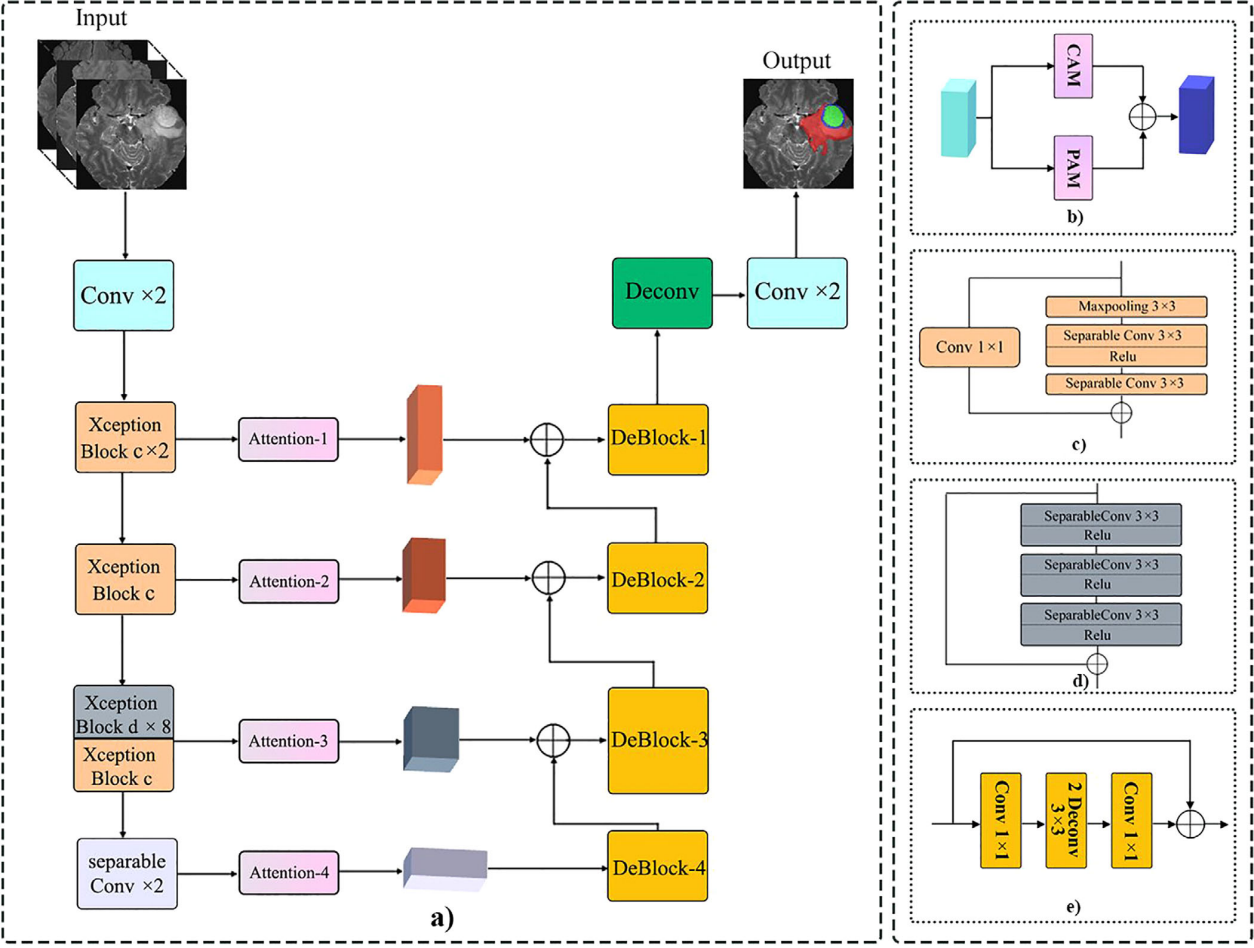
Attention Xception UNet (AXUNet): (a) Model architecture (b) Attention module (c) Xception block utilized in the entry and exit flow (d) Xception block utilized in the middle flow (e) Decoder block. *Note*: The output image was obtained by overlaying output mask on the input image for better visualization.

Augmentations included:

- RandomRotate90,
- Horizontal flip,
- Vertical flip,
- ShiftScaleRotate.

All transformations were synchronously applied to images and corresponding masks.

### 2.3 Model architecture

The proposed Attention Xception U‐Net (AXUNet) integrates:

- Xception encoder backbone,
- Self‐attention module,
- Decoder blocks (DeBlocks).

The overall architecture is illustrated in Figure 2(a), while detailed components are shown in Figure 2(b–e).

#### 2.3.1 Xception backbone

Xception was introduced by F. Chollet.^25^ He took the concept of inception modules^26^ then simplified it to reconstructed an extreme version of it with the help of memory‐efficient separable convolution layers. It consists of three sections: the entry flow, middle flow, and exit flow.

The entry flow initiates with two 3 × 3 convolution layers followed by ReLU activation. Xception blocks, integral to the entry flow, comprise separable convolution, ReLU, another separable convolution, and max pooling. Additionally, a side branch incorporates a single 1 × 1 convolution layer, merging its output with that of the max pooling layer (Figure 2(c)). The entry flow concludes with three Xception blocks. The middle flow consists of eight repeated Xception blocks, each comprising three consecutive ReLU‐separable convolution layers. Unlike the entry flow, these blocks omit the 1 × 1 convolution and max pooling layers, instead integrating input with the output of the final separable convolution (Figure 2(d)). The exit flow utilizes the same Xception block as one of the entry flow's Xception blocks, followed by two separable‐ReLU layers. We used pretrained weights on the Xception backbone of AXUNet. Utilizing pretrained weights provides a better starting point, resulting in faster convergence and improved outcomes.^23^, ^27^

#### 2.3.2 Self‐attention module

The self‐attention module enables efficient contextual encoding through two complementary units: the Pixel Attention Module (PAM), which uses a linear‐complexity kernel attention mechanism, and the Channel Attention Module (CAM), which uses dot‐product‐based channel attention (Figure 2(b)).

- **Pixel Attention Module (PAM)**

In PAM, three 1×1 convolutions produce Q and K with C/8 channels and V with C channels. The three outputs are referred to as query (Q), key (K), and value (V) matrices. Subsequently, Q, K, and V matrices are reshaped as follows:

1. Q (B, C/8, H, W) → Q (B, H×W, C/8)
2. K (B, C/8, H, W) → K (B, C/8, H×W)
3. V (B, C, H, W) → V (B, C, H×W)

where B, C, H, W ∈R denote batch size, number of channel, height, and width, respectively. The formula for PAM's kernel attention is as follows:

(2) $$D\;\left(Q,K,V\right)=\frac{softplus\left(Q\right)softplus{\left(K\right)}^{T}V}{softplus\left(Q\right)\sum_{j}softplus{\left(K\right)}_{i,\;j}^{T}}\;$$

This formula calculates the similarity between Q and K using softplus‐transformed features and nomarlizes the result using the similarity between each query and the sum of the transformed key features across spatial positions. This produces an output matrix with N * C dimensions, where each row represents the aggregated contextual information for a corresponding pixel. By exploiting the associativity of matrix multiplication, the computation avoids explicitly constructing an N × N pixel‐wise attention matrix, thereby reducing computational complexity. The resulting attention features are reshaped to match the input dimensions and multiplied by a learnable parameter (gamma) before being added to the input feature map (Figure 3).

- **Channel Attention Module (CAM)**

**FIGURE 3:**
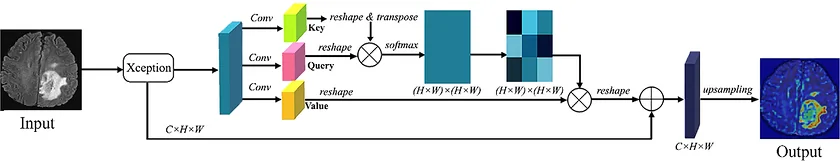
Pixel Attention Module (PAM). *Note*: The output image was obtained by generating Grad‐CAM of PAM module and overlaying it on the input image for better intuition.

The Implementation of CAM module follows the same rules of dot‐product attention. The input X (Batch, Channel, Width, Height) is reshaped to generate Q, V, and K:

1. Q, V = X (B, C, W * H)
2. K = X (B, W * H, C)

Using batch matmul, Q and K are multiplied to produce channel attention (B, C, C). The maximum value along the last dimension is subtracted from the energy matrix before applying softmax along the same dimension to obtain channel attention weights. The resulting attention matrix is multiplied by V to produce the channel attention features. Finally, the attention features are scaled by a learnable parameter (gamma) and added to the input feature map (Figure 4). The outputs of the CAM and PAM modules are added together to form the self‐attention module. In AXUNet, four self‐attention modules are placed between the encoder and decoder.

**FIGURE 4:**
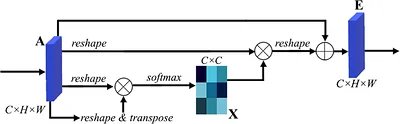
Channel Attention Module (CAM).

#### 2.3.3 Decoder blocks (DeBlocks)

The decoder section consists of four DeBlocks followed by a final deconvolutional layer. Each DeBlock comprises a 1 × 1 convolutional layer, two 3 × 3 deconvolutional layers, and another 1 × 1 convolutional layer, as illustrated in Figure 2(e)). The output of each DeBlock is combined with the attention block and passed to the subsequent DeBlock.

### 2.4 Loss function

Training was performed using a combination of Binary Cross‐Entropy and Dice loss:

(3) $$L=L_{BCE}+L_{Dice}$$

Dice loss is defined as:

(4) $$L_{Dice}=1-\frac{2\sum pg}{\sum p+\sum g}$$

where p denotes sigmoid‐transformed predictions and g ground‐truth labels.

As shown in Equations (3)–(4), the composite loss balances region overlap and voxel‐wise classification.

Losses were computed independently for WT, TC, and ET and averaged.

### 2.5 Model assessment and visualization

Segmentation performance was evaluated using the Dice score Coefficient^28^:

(5) $$DSC=\frac{2TP}{2TP+FP+FN}\;$$

As defined in Equation (5), DSC quantifies spatial overlap between prediction and ground truth.

For interpretability, Grad‐CAM^29^ was applied to:

- The last convolution layer,
- The first self‐attention module,
- The convolution layer of DeBlock3.

The resulting heatmaps are shown in Figure 6, enabling qualitative assessment of model attention distribution.

### 2.6 Implementation details

The model was implemented in PyTorch^30^ and trained on a system equipped with:

- NVIDIA RTX 3090 GPU (24 GB),
- 60 GB RAM.

Optimization was performed using Adam optimizer with learning rate $1\times 10^{-4}$ and cosine annealing scheduler.

Batch size: 64

Total epochs: 40

Model checkpoints were saved based on best validation Dice performance and evaluated on the unseen test partition.

## 3 RESULTS

### 3.1 Ablation study

We first conducted an ablation study to quantify the contribution of each architectural component of the proposed AXUNet. A standard 2D UNet served as the baseline model. This configuration achieved a mean Dice score of 93.26 on the test partition, with class‐wise Dice scores of 91.73 for WT, 86.46 for TC, and 84.81 for ET.

Replacing the UNet encoder with the Xception backbone (Xception‐UNet) resulted in a very small reduction in the overall mean Dice score (−0.02), yielding 93.24 on average. At the subregion level, the Dice score for TC decreased from 86.46 to 83.74 and for ET from 84.81 to 84.44, whereas the WT Dice improved from 91.73 to 92.27.

Subsequently, we augmented the Xception‐UNet with self‐attention modules inserted before each skip connection, resulting in the full AXUNet architecture. This configuration achieved a mean Dice score of 93.73, representing the best overall performance among the ablated variants. The corresponding Dice scores were 92.59 for WT, 86.81 for TC, and 84.89 for ET. Compared with the baseline UNet, AXUNet improved WT and TC segmentation while maintaining ET performance. Relative to Xception‐UNet, AXUNet recovered and slightly exceeded the TC and ET Dice scores.

### 3.2 Comparison with other models

To comprehensively evaluate the proposed AXUNet against other widely used segmentation backbones and attention mechanisms, we compared it with Inception‐UNet, Attention ResUNet (AResUNet), and Attention Gate UNet (AG‐UNet). Quantitative results are summarized in Table 1.

**TABLE 1:** Performance evaluation of AXUNet against other models. (The highest Dice scores are bolded).

		Regional Dice score		
Model	Mean Dice score	WT	TC	ET
UNet	93.26	91.73	86.46	84.81
Xception‐UNet	93.24	92.27	83.74	84.44
Inception‐UNet	90.88	89.36	80.44	82.21
AResUNet	92.80	91.28	85.28	**84.92**
AG‐UNet	90.38	90.19	85.89	83.77
AXUNet (proposed)	**93.73**	**92.59**	**86.81**	84.89

Inception‐UNet achieved a mean Dice score of 90.88, with class‐wise Dice scores of 89.36 for WT, 80.44 for TC, and 82.21 for ET. AResUNet attained a mean Dice score of 92.80 and achieved the highest ET Dice among the comparator models, with scores of 91.28 for WT, 85.28 for TC, and 84.92 for ET. AG‐UNet obtained a mean Dice score of 90.38, with 90.19 for WT, 85.89 for TC, and 83.77 for ET.

AXUNet achieved the highest mean Dice score (93.73) among the compared architectures and provided the best WT and TC Dice scores (92.59 and 86.81, respectively). For ET, AXUNet's Dice score of 84.89 was marginally below AResUNet's 84.92, the highest among the compared models.

### 3.3 Comparative performance visualization

Qualitative comparisons of segmentation outputs are provided in Figure 5, which shows the predictions of six models (UNet, Xception‐UNet, Inception‐UNet, AResUNet, AG‐UNet, and AXUNet) alongside the original MR image and the corresponding ground‐truth labels. WT, ET, and TC regions are color‐coded in red, blue, and green, respectively, to facilitate visual assessment.

**FIGURE 5:**
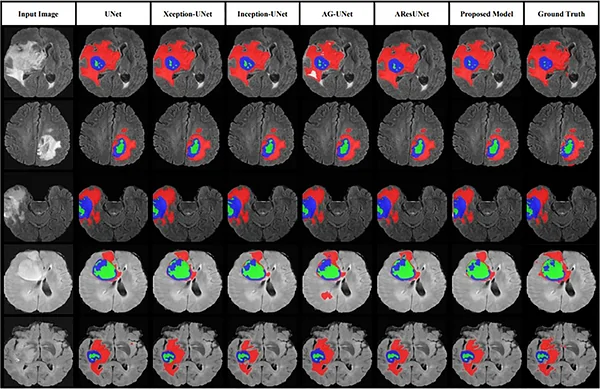
Comparative performance visualization. Color legend: WT (Red+ Blue+ Green), ET (Blue), TC (Green+ Blue).

Across the depicted cases, AXUNet consistently produces segmentations that are closer to the ground truth, particularly in challenging boundary regions and in areas where multiple subregions are in close proximity. In rows 1 and 4, for example, AXUNet more accurately separates adjacent ET and TC regions without spuriously merging them, whereas several comparator models show either over segmentation or boundary blurring that leads to ET–TC fusion. In row 1, AG‐UNet exhibits an abrupt termination of the WT segmentation, resulting in under segmentation of edema. In row 2, all models perform reasonably well; however, Inception‐UNet introduces a narrow, bottleneck‐like artifact in the green TC region.

In row 3, UNet and Xception‐UNet visually appear as strong performers but tend to overestimate the WT region in the posterior edematous area (red). AXUNet reduces this over segmentation while preserving the true extent of the tumor. Row 5 highlights one of AXUNet's main strengths: precise delineation of the interface between WT and TC. Several models either merge these regions or generate substantial errors—UNet, for instance, produces a distant false‐positive WT region in the contralateral hemisphere, and AG‐UNet and Xception‐UNet erroneously fill the space between ET and WT with TC.

### 3.4 Exploring model interpretability with Grad‐CAM

To investigate whether AXUNet's performance gains are aligned with clinically meaningful image features, we conducted a qualitative interpretability analysis using Grad‐CAM. Figure 6 displays Grad‐CAM heatmaps derived from three internal locations of AXUNet—the last convolution layer of the decoder, the first self‐attention module, and the convolutional layer of DeBlock3—for four representative MR images.

**FIGURE 6:**
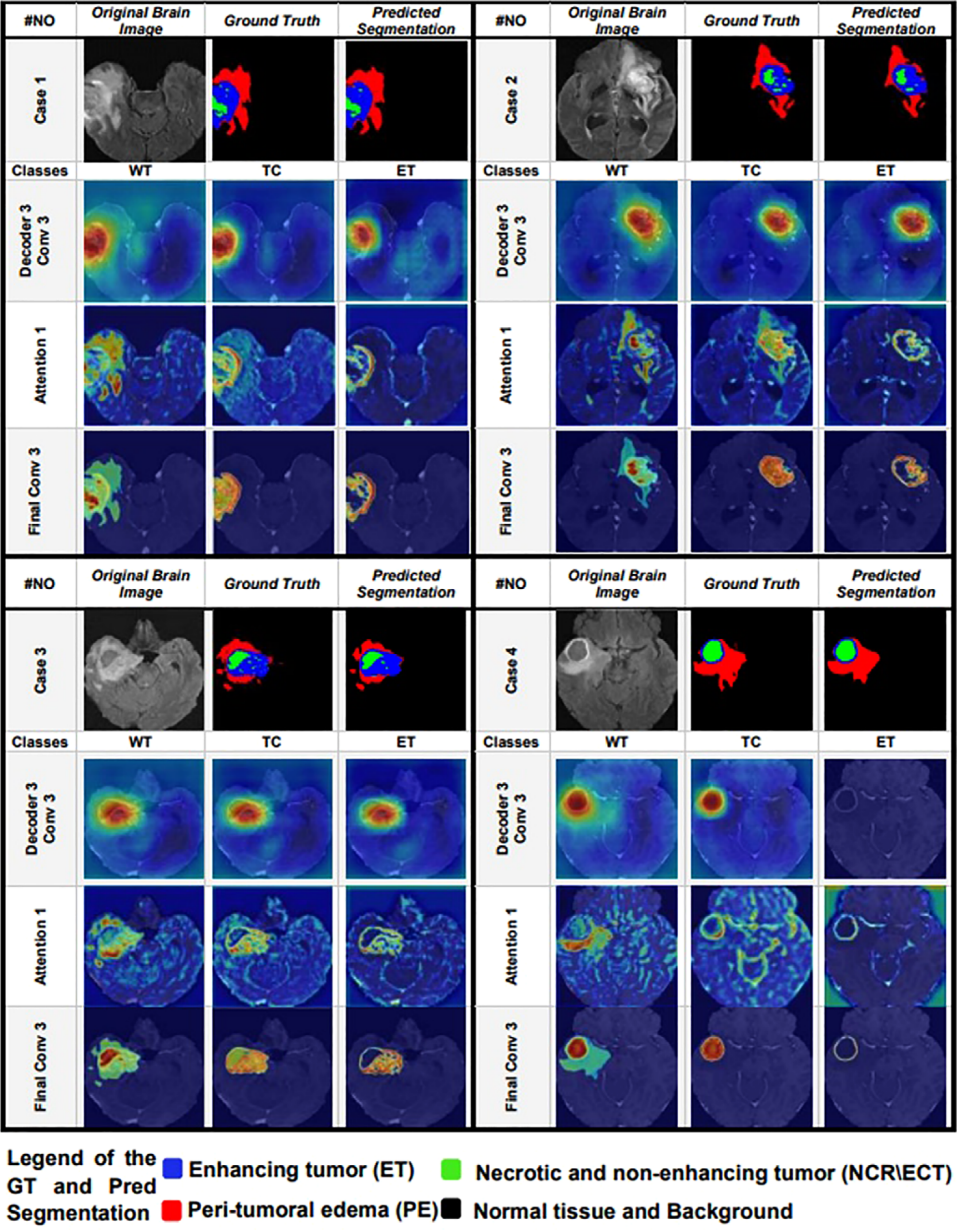
Grad‐CAM visualization.

In these visualizations, regions with higher attention are shown in reddish tones, intermediate attention in yellow–orange, and low attention in green, while areas with negligible contribution remain uncolored. Across cases, AXUNet tends to focus its highest attention on the central parts of the tumor, which generally correspond to TC and ET regions, while also capturing parts of the surrounding edema when predicting WT. When the network models larger extents such as WT, the attention maps spread more broadly but remain centered on the tumor‐bearing hemisphere.

An illustrative example is the ET region in Case 4. In deeper layers, the Grad‐CAM heatmaps are relatively sparse over small ET foci. As we move towards shallower layers (e.g., DeBlock3), the heatmaps become more pronounced over ET.

Another noteworthy observation concerns the model's residual attention to brain sulci and cerebrospinal fluid (CSF) spaces, particularly visible in the heatmap of the first attention module.

Overall, the Grad‐CAM analysis in Figure 6 demonstrates that AXUNet's internal focus is largely aligned with tumor regions, while also revealing specific confounders (e.g., CSF and sulci) in the attention maps.

### 3.5 Independent validation on BraTS 2023: A trustworthiness‐oriented assessment

To further assess the generalization capability and robustness to previously unseen data of AXUNet, we conducted an independent validation using the BraTS 2023 validation dataset on Synapse platform. This evaluation is essential from a Trustworthy AI perspective, as emphasized in recent frameworks such as FUTURE‐AI and the HAITI, which consider out‐of‐distribution performance, robustness, and explainability as critical indicators of reliability in real‐world deployment.

Across the 219 validation subjects, AXUNet achieved Dice scores of 86.29 for WT, 78.53 for TC, and 69.00 for ET. The corresponding Hausdorff95 distances were 32.43 mm, 30.12 mm, and 44.70 mm, respectively. Sensitivity and specificity metrics showed consistent performance across all tumor subregions, indicating a balanced behavior with respect to false negatives and false positives. These results provide evidence that the model maintains relatively stable performance when exposed to heterogeneous multicenter data not seen during training.

#### 3.5.1 Comparison with BraTS 2023 challenge approaches

To contextualize the independent validation results of AXUNet, we compared its performance with two representative approaches reported in the BraTS 2023 literature: the CNN‐based ensemble proposed by Maani et al.^31^ and the winning solution of the BraTS 2023 Adult Glioma challenge.^32^ Maani et al.^31^ employed a weighted ensemble of MedNeXt with deep supervision, SegResNet with deep supervision, and SegResNet without deep supervision, together with task‐specific post‐processing. Their approach ranked third in the final challenge test‐set evaluation. On the Adult Glioma validation leaderboard, the final ensemble achieved Dice scores of 90.63%, 86.27%, and 84.32% for WT, TC, and ET, respectively. AXUNet achieved corresponding scores of 86.29%, 78.53%, and 69.00%, showing lower performance across all three tumor subregions. The smallest difference was observed for WT (4.34 percentage points), whereas larger differences were observed for TC and ET (7.74 and 15.32 percentage points, respectively).

The winning solution of the BraTS 2023 Adult Glioma challenge combined convolutional and transformer architectures within an nnU‐Net–based pipeline augmented by extensive data augmentation strategies, including generative adversarial networks and image registration. This approach achieved validation Dice scores of 90.05%, 86.73%, and 85.09% for WT, TC, and ET, respectively, with corresponding HD95 distances of 14.94, 14.46, and 17.69 mm (32). Compared with this approach, AXUNet demonstrated lower Dice scores across all tumor subregions, particularly for ET segmentation.

Overall, AXUNet achieved lower validation Dice scores than both comparator approaches, with the smallest performance gaps observed for WT and the largest for ET (Table 2). These results should be considered alongside differences in model design and processing strategies: the comparator approaches used model ensembles and additional post‐processing or synthetic data augmentation, whereas AXUNet employed a single CNN‐based architecture with self‐attention, without model ensembling or large‐scale synthetic data augmentation. AXUNet achieved a WT Dice score of 86.29% on unseen validation data, although further improvements are needed, particularly for TC and ET segmentation.

**TABLE 2:** Comparison of lesion‐wise Dice scores (%) on the BraTS 2023 Adult Glioma validation set. (The highest Dice scores are bolded).

Model	Architecture / strategy	WT	TC	ET
AXUNet (ours)	CNN + self‐attention	86.29	78.53	69.00
Maani et al.^31^: MedNeXt‐DS + SegResNet‐DS + SegResNet	CNN– vision transformer ensemble with post‐processing	**90.63**	86.27	84.32
BraTS 2023 winning solution^32^	CNN–transformer ensemble with synthetic data augmentation	90.05	**86.73**	**85.09**

## 4 DISCUSSION

AXUNet addresses a fundamental challenge in glioma segmentation: balancing high‐resolution spatial feature extraction with long‐range global dependency modeling. It does so by integrating an Xception‐based encoder with self‐attention at the skip connections, achieving a mean Dice score of 93.73 on the BraTS 2021 held‐out test partition. The individual components—Xception‐style depthwise separable convolutions and self‐attention—are established techniques; the contribution of this work lies in their specific integration for glioma segmentation and in the evidence, from ablation and independent external testing, that this integration improves robustness and interpretability without relying on ensembling or large‐scale synthetic augmentation. We interpret these results cautiously: segmentation metrics alone, including Dice scores, do not establish clinical benefit. They identify a technically promising architecture whose clinical utility must be tested through dedicated clinician‐reader studies.

The ablation results isolate the contributions of the architectural components. Replacing the UNet encoder with the Xception backbone alone lowered tumor core and enhancing tumor Dice while improving whole tumor Dice, suggesting that separable convolutions capture broad edematous extent effectively but are less able to resolve the finer core and enhancing subregions in isolation. Adding self‐attention at the skip connections recovered tumor core and enhancing tumor performance and slightly exceeded the baseline UNet values, indicating that self‐attention compensates for this degradation and improves feature aggregation specifically at the decoder‐facing skip connections. The gains from self‐attention were observed across all three clinically important subregions rather than being confined to a single class, which reduces the risk of systematically under‐ or over‐segmenting any one compartment. Inserting self‐attention at the skip connections also provides a more selective and interpretable feature‐transfer mechanism than naive concatenation; Grad‐CAM visualizations therefore highlight a component that genuinely filters information reaching the decoder, rather than a post‐hoc addition. However, this does not by itself establish clinical interpretability or utility.

Among the internal comparator models, AXUNet achieved the highest mean Dice score and the best whole tumor and tumor core Dice scores; for enhancing tumor, its Dice score of 84.89 was marginally below AResUNet's 84.92. AXUNet therefore did not outperform every comparator on every subregion, but it provided the most consistent performance across the three regions considered jointly. The qualitative comparisons were consistent with this pattern. AXUNet showed closer boundary agreement with the ground truth, particularly at the whole tumor/tumor core interface and in regions where subregions are adjacent, corresponding to fewer isolated false positives, smaller boundary deviations, and less region fusion than the comparator models. For example, Inception‐UNet produced a narrow tumor core artifact, while UNet and XceptionUNet overestimated whole tumor extent in regions of subtle hyperintensity; AXUNet's attention‐gated skip connections appeared to suppress these errors. These findings are clinically relevant in principle—whole tumor delineation is important for radiotherapy target volume definition, while tumor core and enhancing tumor delineation matter for surgical planning and treatment‐response assessment—but we did not directly measure downstream clinical meaningfulness, and we treat the qualitative reading as illustrative rather than a systematic error‐mode analysis.

Relative to specialized transformer‐based and hybrid architectures reported on BraTS 2021‐style data, such as High‐Resolution Swin Transformer (HRSTNet),^33^ Automated multi‐modal Transformer network (AMTNet),^34^ TransU^2^‐Net,^35^ Clinical Knowledge‐Driven Hybrid Transformer (CKD‐TransBTS),^36^ Extension of the standard 3D U‐Net architecture (E1D3)^37^ and SegTransVAE,^38^ AXUNet's main strength lies in whole tumor segmentation, while its tumor core and enhancing tumor performance trails several of those models^38^, ^39^ (Table 3). Many of those architectures are explicitly designed to capture the fine‐grained contextual information needed for small, irregular enhancing tumor and tumor core subregions. AXUNet's strength appears to be at the tumor‐brain interface rather than in uniformly outperforming specialized models across all subregions. Direct comparisons with previously published methods should be interpreted cautiously because preprocessing pipelines, dataset partitioning, dimensionality, and evaluation protocols vary. No single architecture is optimal for all glioma components; AXUNet offers a balanced and comparatively simple alternative, with modular structure that remains amenable to post‐hoc interpretability analysis.

**TABLE 3:** Comparison of our proposed model and the baseline models on the BraTS 2021 dataset. (The highest Dice scores are bolded).

Model	WT	TC	ET
High‐resolution swin transformer (HRSTNet)^33^	91.90	82.92	87.62
Extension of the standard 3D U‐Net architecture (E1D3)^37^	92.50	89.80	85.60
Two‐Branch network with attention and super‐resolution reconstruction^19^	92.11	90.09	85.13
Automated multi‐modal Transformer network (AMTNet)^34^	92.40	89.50	73.40
TransU^2^‐Net^35^	92.30	86.32	85.88
Swin transformer with enhanced local self‐attention^39^	91.76	88.94	**88.95**
SegTransVAE: hybrid CNN—transformer^38^	90.52	**92.60**	85.48
Proposed	**92.59**	86.81	84.89

Grad‐CAM visual validation indicated that AXUNet focuses largely on FLAIR hyperintensities and the tumor‐bearing hemisphere. For small enhancing tumor foci, attention was relatively sparse in deeper layers and became more pronounced at the shallower decoder stage, suggesting that fine‐scale enhancing components are not fully captured until high‐resolution spatial detail is progressively reintroduced by the decoder. This pattern provides an interpretable rationale for how different encoder‐decoder layers contribute to small‐lesion segmentation. However, AXUNet also showed residual attention to brain sulci and CSF spaces, likely driven by the high signal intensity of CSF on T2‐weighted images. This represents a potential source of attention bias rather than a tumor‐relevant signal. From a safety and robustness standpoint, this is important because it identifies a specific non‐tumoral structure that may contribute to false positives or unstable predictions in cases with pronounced sulcal CSF. Identifying this pattern is a step toward more transparent behavior and enables targeted refinements, but it also underscores that spatial correspondence between model attention and tumor regions does not by itself establish clinical interpretability or clinical utility in the sense of changing a clinician's decision‐making, workflow, or trust. The model also did not replicate some ground‐truth annotation artifacts, suggesting a degree of robustness, although Grad‐CAM activation maps remain relatively coarse and cannot precisely localize feature importance at tumor boundaries.

### 4.1 Limitations and future directions

Several limitations are worth mentioning. Review by a physician identified localized annotation errors in the reference segmentations shown in Figure 7. AXUNet predictions did not reproduce these specific errors. These examples highlight how inaccuracies in reference annotations can affect overlap‐based evaluation metrics, although they do not establish the overall superiority of the model predictions over the reference annotations. The BraTS framework provides a valuable benchmark, but the peritumoral edema region includes both vasogenic edema and infiltrative tumor cells, which limits precise biological characterization. Advanced MRI modalities, such as diffusion‐weighted and perfusion imaging, could improve this distinction and thereby enhance treatment planning and target delineation. The self‐attention mechanism remains computationally intensive. Future iterations could explore “factorized” attention or shallower placement of modules to balance performance with inference speed.^40^ Furthermore, while we conducted independent validation on 219 cases from BraTS 2023, assessing generalizability across diverse clinical protocols and scanner types remains an ongoing necessity. A related limitation is that the BraTS 2023 validation benchmark does not provide a detailed breakdown of the evaluated cases by glioma grade; the proportion of low‐grade (LGG) and high‐grade (HGG) glioma subjects within the 219 validation cases is therefore unknown. Since AXUNet was trained on a mixed LGG/HGG cohort from BraTS 2021, and tumor subregions such as ET, TC, and WT differ substantially in prevalence and morphology between LGG and HGG cases, it is difficult to determine whether the external performance reported in Section 3.5 reflects balanced generalization across grades or is disproportionately influenced by one grade category. Future work should include grade‐stratified independent validation to resolve this. Another limitation is that AXUNet is a 2D architecture, which may not fully exploit three‐dimensional contextual information available across adjacent MRI slices. Future work may investigate 3D or hybrid 2.5D extensions while preserving computational efficiency. Finally, Grad‐CAM provides only partial interpretability, and the current study does not include clinician‐reader evaluation, fairness analysis, or robustness testing beyond the external BraTS cohort. Future work should incorporate more precise interpretability techniques such as occlusion sensitivity analysis and should explicitly evaluate clinical utility rather than inferring it from segmentation metrics alone.

**FIGURE 7:**
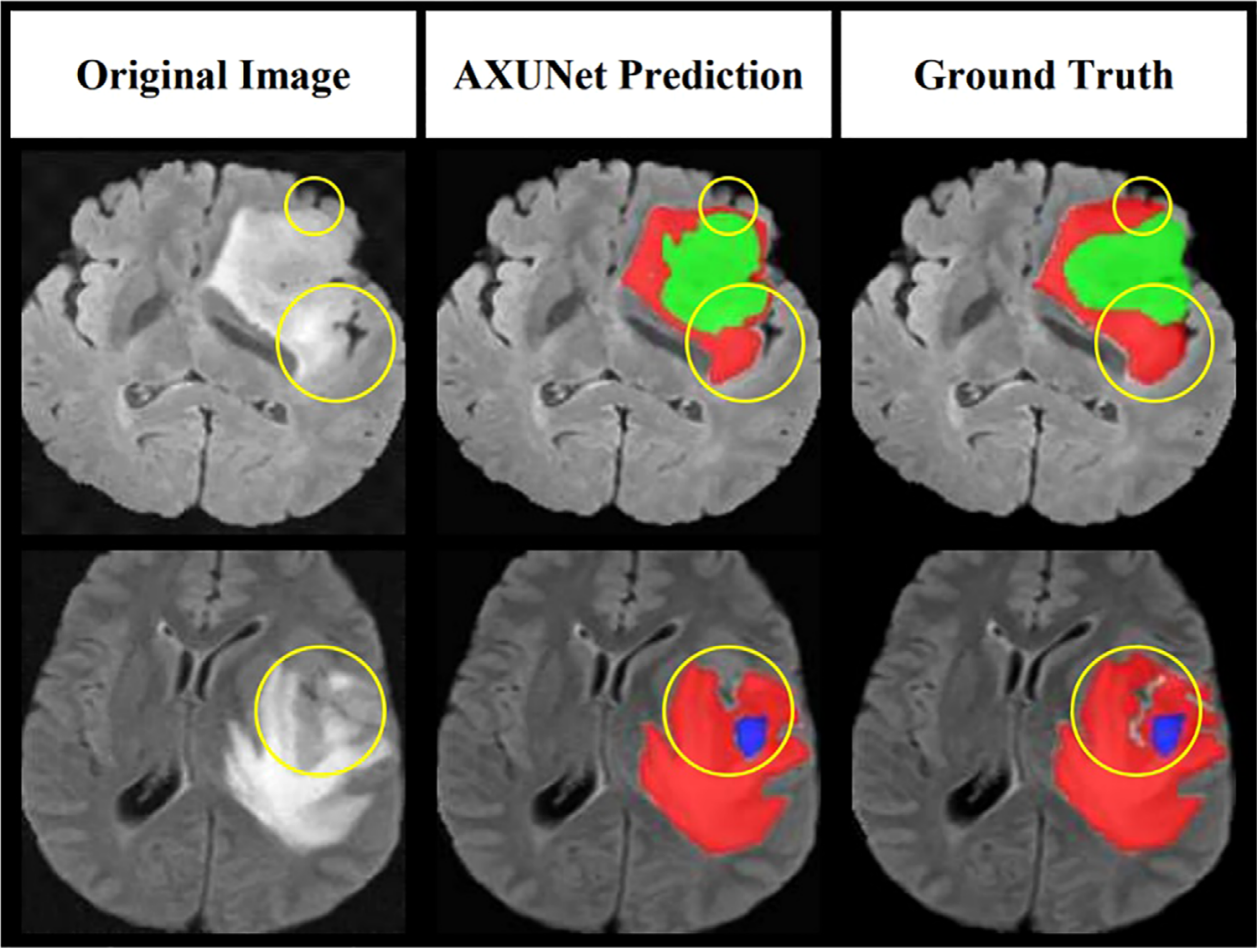
Examples of physician‐identified errors in reference annotations. Original MRI images (left), AXUNet predictions (middle), and ground‐truth annotations (right) are shown. Yellow circles highlight corresponding regions in which physician review identified annotation errors, allowing comparison with the model predictions.

## 5 CONCLUSION

AXUNet combines an Xception encoder with self‐attention to curb information redundancy in skip connections and sharpen tumor‐focused feature transmission, outperforming standard UNet and several attention‐based variants on glioma segmentation. Grad‐CAM analysis confirms that its internal focus is largely tumor‐aligned, while also surfacing a CSF‐related attention bias that highlights the gap between spatial correspondence and genuine clinical interpretability. Together, these results position AXUNet as an efficient, modestly interpretable segmentation framework, with robustness on small enhancing‐tumor regions and validation across multi‐institutional and post‐operative cohorts remaining open directions for clinical translation.

## AUTHOR CONTRIBUTION

**Dornaz Mazinani**: Software, formal analysis, data curation, visualization, investigation. **Farzan Moodi**: Writing—original draft, methodology, data curation, investigation, software, visualization. **Hanieh Mobarak Salari**: Data curation, investigation, resources. **Hamidreza Saligheh Rad**: Conceptualization, supervision, project administration, methodology, writing—review & editing, resources. **Fereshteh Khodadadi Shoushtari**: Supervision, writing—review & editing, software, formal analysis, visualization, methodology. **Sedigheh Sina**: Writing—review & editing, methodology. **Gelareh Valizadeh**: Supervision, methodology, software, writing—review & editing.

## CONFLICT OF INTEREST STATEMENT

The authors declare no conflicts of interest.

## ETHICAL APPROVAL

This study used publicly available, de‐identified data from Brain Tumor Segmentation (BraTS) 2021 and 2023 datasets (Synapse ID: syn51156910) were used for training and evaluation under the CC BY‐NC 4.0 license for non‐commercial research and did not involve direct recruitment of human participants or collection of identifiable personal data.

## Data Availability

Brain Tumor Segmentation (BraTS) 2021 and 2023 datasets (Synapse ID: syn51156910) were used for training and evaluation under the CC BY‐NC 4.0 license for non‐commercial research.
